# Inhibition of Angiogenesis by Treatment with Cold Atmospheric Plasma as a Promising Therapeutic Approach in Oncology

**DOI:** 10.3390/ijms21197098

**Published:** 2020-09-26

**Authors:** Lyubomir Haralambiev, Ole Neuffer, Andreas Nitsch, Nele C. Kross, Sander Bekeschus, Peter Hinz, Alexander Mustea, Axel Ekkernkamp, Denis Gümbel, Matthias B. Stope

**Affiliations:** 1 Department of Trauma, Reconstructive Surgery and Rehabilitation Medicine, University Medicine Greifswald, Ferdinand-Sauerbruch-Straße, 17475 Greifswald, Germany; oleneuffer@aol.de (O.N.); an124100@uni-greifswald.de (A.N.); nk144013@uni-greifswald.de (N.C.K.); peter.hinz@med.uni-greifswald.de (P.H.); ekkernkamp@ukb.de (A.E.); denis.guembel@uni-greifswald.de (D.G.); 2 Department of Trauma and Orthopaedic Surgery, BG Klinikum Unfallkrankenhaus Berlin gGmbH, Warener Straße 7, 12683 Berlin, Germany; 3 ZIK plasmatis, Leibniz Institute for Plasma Science and Technology (INP), Felix-Hausdorff-Straße 2, 17489 Greifswald, Germany; sander.bekeschus@inp-greifswald.de; 4 Department of Gynecology and Gynecological Oncology, University Hospital Bonn, Venusberg-Campus 1, 53127 Bonn, Germany; alexander.mustea@ukbonn.de (A.M.); matthias.stope@ukbonn.de (M.B.S.)

**Keywords:** apoptosis, cell migration, cold atmospheric plasma, endothelial cells VEGF

## Abstract

Background: Cold atmospheric plasma (CAP) is increasingly used in the field of oncology. Many of the mechanisms of action of CAP, such as inhibiting proliferation, DNA breakage, or the destruction of cell membrane integrity, have been investigated in many different types of tumors. In this regard, data are available from both in vivo and in vitro studies. Not only the direct treatment of a tumor but also the influence on its blood supply play a decisive role in the success of the therapy and the patient’s further prognosis. Whether the CAP influences this process is unknown, and the first indications in this regard are addressed in this study. Methods: Two different devices, kINPen and MiniJet, were used as CAP sources. Human endothelial cell line HDMEC were treated directly and indirectly with CAP, and growth kinetics were performed. To indicate apoptotic processes, caspase-3/7 assay and TUNEL assay were used. The influence of CAP on cellular metabolism was examined using the MTT and glucose assay. After CAP exposure, tube formation assay was performed to examine the capillary tube formation abilities of HDMEC and their migration was messured in separate assays. To investigate in a possible mutagenic effect of CAP treatment, a hypoxanthine-guanine-phosphoribosyl-transferase assay with non malignant cell (CCL-93) line was performed. Results: The direct CAP treatment of the HDMEC showed a robust growth-inhibiting effect, but the indirect one did not. The MMT assay showed an apparent reduction in cell metabolism in the first 24 h after CAP treatment, which appeared to normalize 48 h and 72 h after CAP application. These results were also confirmed by the glucose assay. The caspase 3/7 assay and TUNEL assay showed a significant increase in apoptotic processes in the HDMEC after CAP treatment. These results were independent of the CAP device. Both the migration and tube formation of HDMEC were significant inhibited after CAP-treatment. No malignant effects could be demonstrated by the CAP treatment on a non-malignant cell line.

## 1. Introduction

Cancer is one of the most common diseases worldwide [1] and represents a major challenge for the healthcare system [2]. The treatment costs caused by malignant neoplasms in Germany rose from 11.7 billion euros in 2002 to 19.9 billion euros in 2015, illustrating the growing social importance of cancer [3].

The differentiation and spread of malignant tumors at the time of diagnosis is crucial for the treatment so that knowledge of the tumor extent is essential for the prognosis of cancer [4]. Cancer can often be treated by the surgical removal of the tumor, chemotherapy, radiation, or a combination of these treatment options [5]. However, curative therapy is often not possible for many types of cancers due to a lack of surgical resectability, tumor remission, or possible metastases. Chemotherapeutic agents are usually not specific for tumor cells, which is why severe adverse drug reactions often occur [5,6]. In addition, even if the initial response to chemotherapy is good, development of resistance plays an important role in therapy failure and tumor recurrence [7].

Regardless of the type of cancer, cancer cells of solid tumors lose their organ-specific functionality and destruct the surrounding healthy tissue through uncontrolled and invasive growth [8]. Tumor cells can invade surrounding blood and lymphatic vessels spread throughout the body, and form metastases in other organs [9]. Therefore, tumor-associated angiogenesis is a critical issue in anti-oncological therapy. This complex process is controlled by soluble angiogenic factors, such as VEGF (vascular endothelial growth factor), bFGF (basic fibroblast growth factor), PDGF (platelet derivated growth factor), angiopoietin, prostaglandins and other proangiogenetic factors [10,11]. Their production by tumor cells is primarily induced in hypoxia [12,13]. When VEGF-A, in particular, binds to the tyrosine kinase receptors KDR (kinase insert domain receptor), and Flt-1 (fms related tyrosine kinase 1) in endothelial cells, these become activated [14,15], and endothelial surface proteins, such as PECAM 1 (platelet endothelial adhesion molecule 1), and VE-Cadherin (vascular endothelial cadherin) are induced [16,17,18], as well as VEGF [19]. As a result, endothelial cells gain mobility, can overcome physical barriers and can migrate into the interstitium [20]. Elevated concentrations of VEGF also lead to a local increase in blood vessel permeability [15]. The release of angiogenic factors exerts a chemotactic effect on endothelial cells so that the sprouting of the cells occurs specifically along the VEGF gradient [21,22]. Such vascular networks are characteristic of tumor angiogenesis, which is primarily mediated by VEGF secreted by cancer cells [19,23,24,25]. As a result, intense vascularization is a characteristic of aggressively growing tumors and facilitates the formation of metastases [26]. Antibodies directed against VEGF have been used in cancer therapy for several years to inhibit tumor progression and subsequent metastasis [24,27]. VEGF-specific antibodies, such as bevacizumab, can reduce vascularization of tumors; however, a variety of side effects and the development of resistance to these antibodies limit their use. In order to improve and expand the existing treatment options in oncology, the application of cold atmospheric plasma (CAP) is becoming increasingly important.

CAP is a highly reactive gas mixture consisting of ions, neutral particles, such as atoms and molecules, radicals, free electrons, photons, and electromagnetic radiation [28,29]. The medical application of CAP has already been established in wound therapy, the sterilization of medical implants and devices, and the disinfection of air [30,31]. Another promising application of CAP is its use in the field of oncology, since an anti-proliferative effect of CAP on cancer cells of various cancers has already been demonstrated [32,33,34,35]. It is believed that, among other effects, the induction of apoptosis is the main reason for the anti-proliferative effect of CAP on cancer cells [34]. Different components of CAP, such as oxygen and nitrogen species—e.g., hydrogen peroxide—are responsible for the induction of programmed cell death [36,37,38]. 

The influence of CAP on endothelial cell proliferation and thus on tumor-associated angiogenesis is underexplored and hence investigated in this study.

## 2. Results

**Figure 1:**
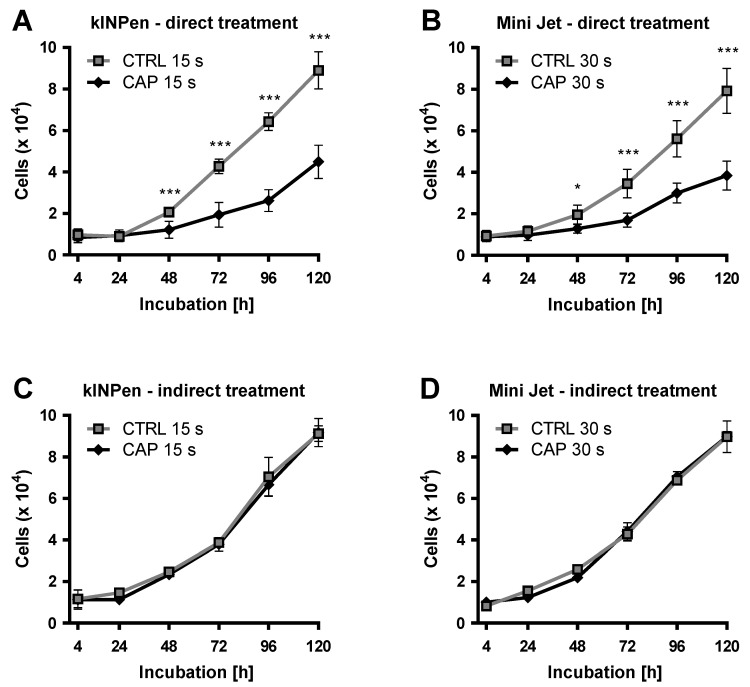
Curbing the HDMEC growth after direct and indirect CAP-treatment. HDMEC were treated with cold atmospheric plasma (CAP) generated by a kINPen (**A**) or Mini Jet (**B**) device. HDMEC were treated with CAP-treated cell culture media by kINPen (**C**) and by Mini Jet (**D**). As control (CTRL) cells were treated with the carrier gas argon. The number of viable cells was examined at the indicated times. Four independent experiments were performed. Results are presented as means ± SD. Two-way RM ANOVA was performed. Student’s *t*-test was used with the following significance levels. The results of the Sidak’s post-hoc tests were indicated as follows: *p* < 0.05 (*), *p* < 0.001 (***).

Human Dermal Microvascular Endothelial Cells (HDMEC) were directly treated with cold atmospheric plasma (CAP) and compared to cells treated with argon carrier gas only (mock control). Direct treatment of HDMEC was performed with the two different CAP devices: kINPen (neoplas tools, Greifswald, Germany) and Mini Jet (Heuermann HF-Technik GmbH). Anti-proliferative effects were observed with both CAP devices, which led to statistically significant growth inhibition from an incubation period of 48 h (Figure 1A,B). Growth inhibitory effects were treatment time-dependent (data not shown). For both CAP devices, a treatment time was determined, in which the cell number of CAP-treated cells was reduced to approximately half that of the control group treated with argon. In the case of kINPen, this was achieved by 15 s treatment time. After 120 h, the cell number of CAP-treated cells was 50.3 ± 6.0% compared to the control group. Treatment time had to be increased for the Mini Jet to obtain an anti-proliferative effect comparable to the treatment with kINPen. This was achieved by a 30 s treatment time. After 120 h, the cell number of CAP-treated cells showed 49.8 ± 7.8% compared to the control group. These treatment times were applied in all subsequent experiments.

The anti-proliferative effect of CAP treatment on cells could possibly be influenced by components of the cell culture medium. In particular, chemical modifications of these components—e.g., amino acids—could influence cellular metabolism. To investigate this indirect influence, untreated HDMEC were incubated with CAP- and argon-treated cell culture media for 120 h. The number of viable cells was examined 4, 24, 48, 72, 96, and 120 h after treatment. For both CAP devices, no statistically significant difference in growth between the indirectly treated HDMEC compared to control cells was found (Figure 1C,D). 

**Figure 2:**
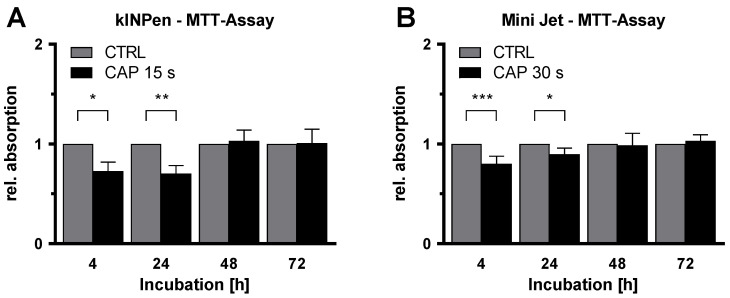
Inhibition of metabolism of endothelial cells after CAP-treatment. HDMEC were treated with cold atmospheric plasma (CAP) generated by a kINPen (**A**) or Mini Jet (**B**) device and incubated up to 72 h. MTT assays were performed at the indicated time points. Absorption was measured and normalized to the number of viable cells. Data were normalized to the argon-treated control cells and are presented as mean ± SD. At least 5 independent experiments, each with three replicates, was performed. Two-way RM ANOVA was used before normalization. The results of the Sidak’s post-hoc tests are indicated as follows: *p* < 0.05 (*), *p* < 0.01 (**), *p* < 0.001 (***).

After inhibition of HDMEC proliferation after CAP-treatment had been demonstrated, the influence of CAP on cellular metabolism was examined using MTT assay. This is an established assay to assess the general metabolic activity of viable cells. HDMEC were treated with CAP or argon gas and analyzed by MTT assay after 4, 24, 48, and 72 h of incubation. For the first two measurement points, a statistically significant reduction in cell metabolism was detected after treatment with both CAP devices (Figure 2). This reduction was more pronounced after CAP treatment with the kINPen than after treatment with the Mini Jet. After 4 h, metabolic activities in CAP-treated HDMEC were reduced to 72.7% ± 9.0% (*p* = 0.041) (kINPen) and 80.2% ± 7.4% (*p* < 0.001) (Mini Jet) compared to the control. After 24 h, reductions to 70.2% ± 8.2% (*p* = 0.002) (kINPen) and 89.7% ± 6.1% (*p* = 0.040) (Mini Jet) were obtained. The inhibitory effect of CAP treatment was not statistically significant after longer incubation periods of 48 and 72 h.

**Figure 3:**
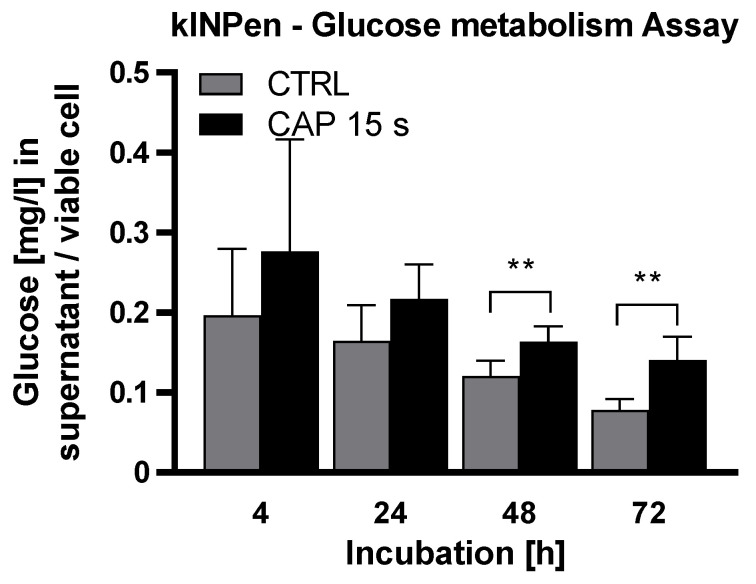
CAP treatment reduces glucose metabolism in HDMEC. HDMEC were treated with cold atmospheric plasma (CAP) generated by a kINPen and incubated up to 72 h. The glucose concentration in the culture medium was measured at the indicated time points and normalized to the number of viable cells. Six independent experiments were performed, each with three replicates. The experiment was statistically evaluated with a two-way RM ANOVA. The results of the Sidak’s post-hoc tests are indicated as follows: *p* < 0.01 (**).

The cell glucose uptake after CAP treatment was measured to further investigate the effects of CAP treatment on cell metabolism. For this purpose, the glucose concentration in the culture medium was determined 4, 24, 48 and 72 h after the treatment. The measured concentration was normalized to the number of living cells. The glucose concentration of the cell culture medium per cell differed significantly (F (1, 10) = 9.897) between CAP and the control (Figure 3). Then, 48 and 72 h after treatment the glucose concentration per cell was significantly (48 h: *p* = 0.009, 72 h: *p* = 0.007) higher in the CAP treated group (48 h: CAP: 0.1634 ± 0.0167, CTRL: 0.1206 ± 0.0168, 72 h: CAP: 0.1405 ± 0.0257, CTRL: 0.0783 ± 0.0116).

**Figure 4:**
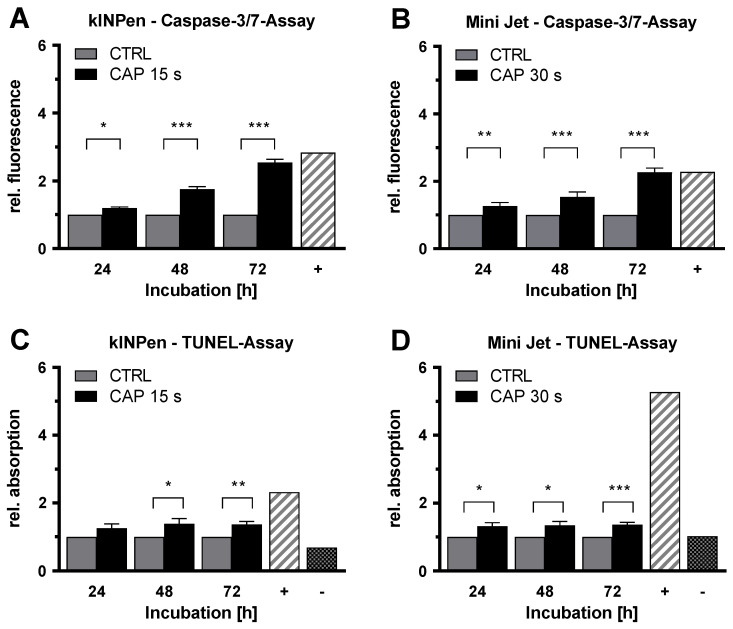
CAP treatment increases the apoptotic signals independent of CAP device. Endothelial cells were treated with cold atmospheric plasma (CAP) or argon carrier gas as control (CTRL), released by the kINPen (**A**,**C**) or Mini Jet (**B**,**D**) device. Caspase-3/7 and TUNEL assays were performed at the indicated times. Positive controls (+) were carried out by treatment with Cycloheximide (**A**,**B**) or nuclease treatment (**C**,**D**). For TUNEL assay, a negative control (-) without labeling was performed. Five independent experiments were performed. The results were normalized to control cells and are presented as mean ± SD. All experiments were statistically evaluated with two-way RM ANOVA with Sidak’s post hoc tests before normalization. The following significance levels were defined: *p* < 0.05 (*), *p* < 0.01 (**), *p* < 0.001 (***).

To verify whether the anti-proliferative effect of CAP treatment was based on the induction of apoptotic processes, caspase-3/caspase-7 activity assays (caspase-3/7 assay) were performed. HDMEC were treated with CAP or carrier gas argon as a control and incubated for 24 h, 48 h, and 72 h. At each time, CAP-treated HDMEC demonstrated a significantly increased caspase-3 and caspase-7 signal, with lower apoptosis induction after Mini Jet treatment than after kINPen treatment (Figure 4A,B). The activity of both caspases increased with prolonged incubation time. After 72 h, the signal strengths of activated caspase-3 and caspase-7 in CAP-treated HDMEC were 2.5 fold (*p* < 0.001) (kINPen) and 2.3 fold (*p* < 0.001) (Mini Jet) compared to control.

TUNEL assays confirmed the findings obtained by the caspase-3/7 assays. HDMEC were treated with CAP or argon gas and incubated for 24, 48, and 72 h. For treatment with both CAP devices, an increased signal of DNA fragmentation was observed (Figure 4C,D). The TUNEL signals increased 1.4 fold (*p* = 0.006) (kINPen) and 1.4 fold (*p* < 0.001) (Mini Jet) 72 h after CAP-treatment.

**Figure 5:**
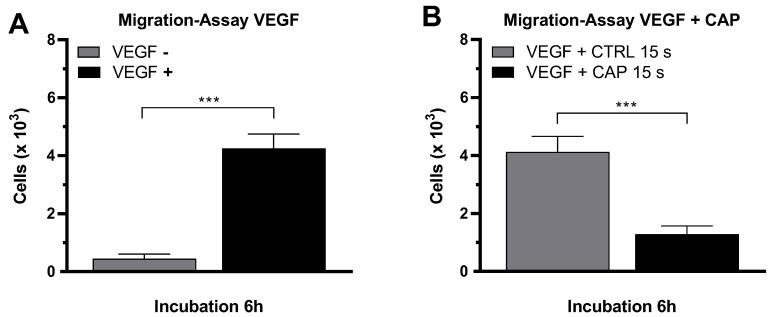
CAP inhibits the migration of endothelial cells. HDMEC were seeded in cell culture inserts with 8 µm pores. A VEGF gradient was established over the membrane. The number of migrated cells was determined after 6 h of incubation (**A**). HDMEC were treated with CAP or argon carrier gas before performing the migration assay with VEGF (**B**). Five experiments were performed. The results are presented as means ± SD. All experiments were statistically evaluated with the Student’s *t*-test: *p* < 0.001 (***).

The migration of endothelial cells into surrounding tissues is a key event of angiogenesis and is a directed process that can be stimulated primarily by the angiogenic factor VEGF. After it was shown that CAP-treatment inhibits the proliferation of HDMEC, the influence of CAP on the migration behavior of HDMEC was also investigated. For this purpose, a migration assay was established in which HDMEC migrated through the membrane of a FluoroBlok Transwell insert (Corning, New York, NY, USA; pore size 8 µm). The number of migrated cells was determined by fluorescence microscopy. In the presence of VEGF, the migration of HDMEC was statistically significantly increased. The number of migrated HDMEC increased 9.5-fold (*p* < 0.001) after an incubation of 6 h (VEGF–negative: 449 ± 157, VEGF–positive: 4250 ± 495 (Figure 5A).

Following this, the effect of CAP treatment on the migration behavior of HDMEC was examined in this setup. For this purpose, the HDMEC were treated with CAP or argon gas (control) before the migration assay. To stimulate the migration of HDMEC, VEGF was present in both approaches. After an incubation period of 6 h, a statistically significant migration inhibition in CAP-treated HDMEC was detected (Figure 5B). The number of migrated CAP-treated HDMEC after 6 h was 3.2-fold lower than the control cells (*p* < 0.001) (CAP: 1287 ± 286, Control: 4129 ± 533). 

**Figure 6:**
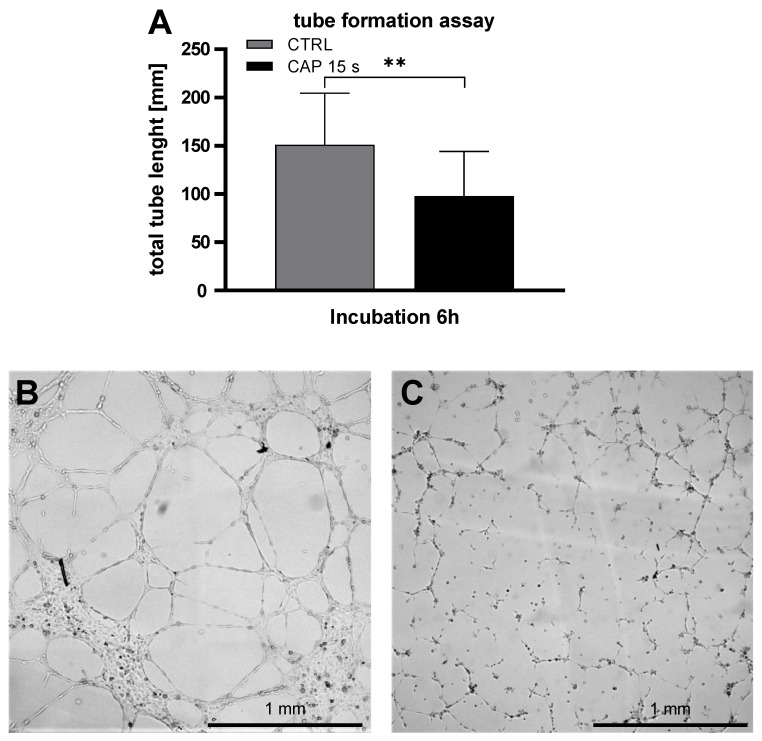
CAP inhibits the tube formation of HDMEC. HDMEC were treated with CAP or carrier gas Argon and seeded in µ-slides pre-coated with Matrigel/Medium. After an incubation period of 6 h, pictures were captured and the total tube length were analyzed with imageJ. The total tube length of the control treated HDMEC was significantly longer than the CAP treated cells (**A**). Six independent experiments were performed. Data are given as means ± SD. The data were statistically evaluated with the paired *t*-test: *p* < 0.01 (**). (**B**,**C**) show representative capture of control-(**B**) or CAP-(**C**) treated cells.

To investigate the influence of CAP on the ability of HDMEC to form tubes in Matrigel, tube formation assays was performed. The total tube length after an incubation period of 6 h was analyzed. The total tube length of CAP treated HDMEC was significantly shorter than the control treated cells (*p* = 0.005, CTRL: 151.1 ± 48.5, CAP: 97.9 ± 42.2) (Figure 6).

**Figure 7:**
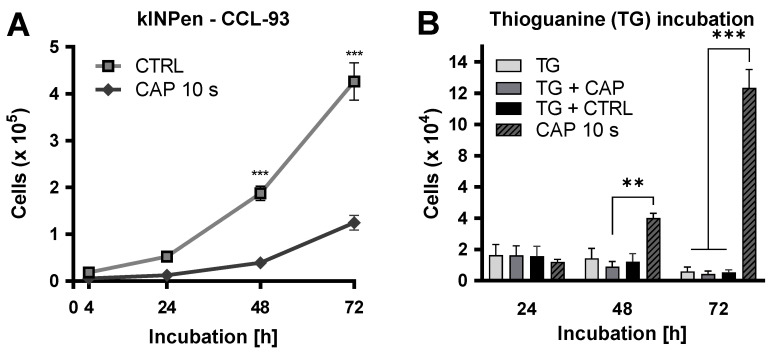
CAP treatment does not lead to mutagenicity. CCL-93 cells were treated with CAP or carrier gas. Growth kinetics were performed over 72 h (**A**). CAP- and argon-treated cells were propagated in medium containing 6-thioguanine (TG) (**B**). Number of viable cells was measured after 24, 48, and 72 h. Untreated cells propagated in medium containing TG and CAP treated cells propagated in medium without TG were used as controls. Three independent experiments were performed. Data are given as means ± SD. All experiments were statistically evaluated with two-way RM ANOVA with Sidak’s post hoc tests. The following significance levels were defined: *p* < 0.01 (**), *p* < 0.001 (***).

To examine a possible mutagenic effect of the CAP treatment, a hypoxanthine-guanine-phosphoribosyl-transferase (HPRT) assay was carried out [39]. For this purpose, CCL-93 cells were treated with CAP or the carrier gas. Growth kinetics were carried out over 72 h. This showed a significant reduction in the number of cells after CAP treatment after 48 (*p* < 0.001) and 72 h (*p* < 0.001). After, treatment cells were incubated in medium containing 6-thioguanine (TG). TG should inhibit cell growth in non-mutated cells (Figure 7). 

## 3. Discussion

A high degree of vascularization is required to satisfy the high oxygen and nutrient requirement of tumor cells and is the most critical prerequisite for rapid and aggressive tumor growth [26]. Therefore, tumor-associated angiogenesis is a major factor in the initiation and progression of malignant tumors [25]. Tumor cells can induce angiogenesis by secreting cytokines, such as VEGF, thereby stimulating endothelial cells to proliferate and migrate [25,26,40,41]. This confirms the cell culture model established in the present study and demonstrated that the presence of VEGF significantly increased the invasiveness of HDMEC.

In addition to radiation, chemotherapy and surgical debridement, the treatment of malignant tumors can be complemented by the inhibition of tumor-induced angiogenesis [26,42]. Several drugs are currently available to suppress angiogenesis, such as the VEGF-specific antibody, bevacizumab. However, bevacizumab therapy can cause severe systemic side effects or development of resistance, which may restrict its application [43,44,45,46]. Against this background, CAP treatment can be considered a new alternative in anti-oncological therapy. It has been shown that CAP treatment can inhibit both the proliferation and the cell mobility of in vitro propagated tumor cells [35,47,48,49].

CAP treatment of HDMEC inhibited their migration, depending on the duration of treatment and the CAP device. The inhibitory effects of the CAP treatment also have a direct influence on the tube formations of HDMEC. This suggests that CAP treatment can inhibit not only the tumor cells directly but also tumor-associated cells, such as endothelial cells. One such example is the inhibitory effect of CAP on fibroblasts [50,51]. These effects were also shown in the current study. In contrast to systemic therapies, CAP only has a locally limited effect. Other studies also indicate that CAP treatment has fewer systemic side effects than conventional therapies [52,53,54]. Another favorable property of CAP is that no resistance to CAP treatment has been observed in previous studies [52,55]. Over the entire study, no reduced CAP efficacy could be detected in the present study. Indirectly mediated anti-proliferative CAP effects have not been demonstrated. A possible explanation can be explained as the inclusion of ascorbic acid in the cell medium. This is known to reduce reactive oxygen species (ROS) and thus prevents apoptosis [56] and thus can weaken the CAP effects [51]. In addition to the migration-inhibiting effect of CAP on endothelial cells, an anti-proliferative effect on human microvascular endothelial cells was also demonstrated for the first time for both CAP devices. The selection of the appropriate treatment time is crucial, since short treatment times have hardly any anti-proliferative effects, but a sharply increased CAP treatment can damage adjacent tissue [57]. Similar to the use of pharmacological compounds, a dose–response relationship should, therefore, be defined for CAP treatment. It is known that the anti-proliferative effect of both CAP sources depends on the treatment time [34]. However, device-specific parameters are also conceivable with which a dose–response relationship may be defined, such as the voltage between the electrodes and the flow rate of the carrier gas [58]. These findings help to understand why different treatment times with both CAP devices were required to achieve an almost identical anti-proliferative effect. With the Mini Jet, the gas flow rate (1.5 L/min) was only half as high as with the kINPen (3 L/min), while the treatment time required to achieve a comparable effect was twice as long. The carrier gas and the CAP device itself represent other critical parameters on which the composition of the cold atmospheric plasma depends [55]. CAP includes charged particles and radicals as well as electromagnetic radiation [59,60]. Due to this heterogeneity of CAP composition, treatment parameters for each CAP source must be defined individually for each cell type and in clinical practice for each tumor type [34].

The anti-proliferative effect of CAP in tumor cells is mainly due to the increase in intracellular concentrations of ROS and nitrogen species (RNS) with corresponding cell biological consequences [61]. The glucose assay showed inhibition of the glucose metabolism of the HDMEC after CAP treatment, since the consumption of glucose by the CAP-treated cells was significantly suppressed up to 72 h after the treatment. Additionally, the MTT assay demonstrated that the metabolic activity [62,63] of endothelial cells was reduced up to 24 h after CAP treatment. However, the metabolism of HDMEC does not appear to be permanently restricted, as the surviving HDMEC were able to regenerate and showed a metabolic activity comparable to that of the control group after 48 h. In other endothelial cells (HUVEC), the restoration of physiological cell metabolism after CAP treatment has already been shown [64]. Comparable metabolic CAP effects have also been described in squamous epithelium cells and fibroblasts [65,66]. This demonstrated regeneration of cell metabolism could not be observed in the tumor cells examined [58,66,67,68]. The different effects of CAP on the metabolic activity of different cell types may be explained by cell type-specific redox-protective mechanisms. In contrast to non-malignant cells, tumor cells are believed to be highly susceptible to oxidative stress [58,65,66]. It is known that oxidative stress triggers apoptosis in epithelial cells [69]. Although CAP-induced apoptosis has been demonstrated in various tumor entities [34,35,70,71], the effect in endothelial cells is still largely unexplored. Analysis of apoptotic processes revealed both increased DNA fragmentation and increased activity of caspase 3 and caspase 7 in HDMEC directly treated with CAP. Indirectly mediated anti-proliferative CAP effects were not observed in HDMEC. 

Discussing the use of CAP for medical treatment, the question of the safety of this procedure always arises. This is particularly relevant for a possible intraoperative use of CAP, since non-malignant areas of the surrounding tissue can also be affected by the CAP treatment. 

The reports by other authors about the impact of CAP on normal, non-malignant cells show no undesirable effects to date [72]. The control tests of this study with a non-malignant cell line (CCL-93) also showed that the CAP treatment does not have any mutagenic effects on normal cells [73].

## 4. Materials and Methods

### 4.1. Cell Culture

Human endothelial cell line HDMEC (Promocell, Heidelberg, Germany) were propagated in a humidified atmosphere at 5% CO_2_ and 37 °C in ECGM-MV medium (Endothelial Cell Growth Medium MV 2; Promocell, Heidelberg, Germany) with the following medium supplements: Fetal Calf Serum 0.05 mL/mL; Epidermal Growth Factor 5 ng/mL, Basic Fibroblast Growth Factor 10 ng/mL; Insulin-like Growth Factor (Long R3 IGF) 20 ng/mL; Vascular Endothelial Growth Factor 165 0.5 ng/mL; Ascorbic Acid 1 μg/mL; Hydrocortisone 0.2 μg/mL. Further, 1% *v/v* penicillin/streptomycin (PAN Biotech, Aidenbach, Germany) was then added to this medium.

CCL-93 cells (ATCC, Manassas, VA, USA), the non-malignant fibroblast cells of cricetulus griseus, were propagated in a humidified atmosphere at 5% CO_2_ and 37 °C in DMEM containing 4.5 g/L glucose with 10% *v/v* FCS and 1% *v/v* penicillin/streptomycin (all from PAN Biotech, Aidenbach, Germany).

### 4.2. Proliferation Assay after CAP Exposure

Cell growth was determined after 4, 24, 48, 72, 96, and 120 h using a CASY cell counter and analyzer model TT (Roche Applied Science, Mannheim, Germany) with a 150 µm capillary. For this purpose, 1 × 10^4^ endothelial cells were suspended in 200 µL culture media and treated with CAP or carrier gas argon (control group) for 10 s (kINPen) or 30 s (Mini Jet). After treatment, the cell suspension was transferred to another 24-Well cell culture plate. The treatment well was rinsed with 200 µL fresh media, which was also transferred to the culture plate. Then, 800 µL fresh media was added, and cells were incubated in a humidified atmosphere at 5% CO_2_ and 37 °C. Cell count was determined by suspending the cells by trypsin/EDTA (Ethylenediaminetetraacetic acid) treatment and diluting 100 µL cell suspension in 10,000 µL CASYton (Roche Applied Science, Mannheim, Germany). The measurement was performed three times with 400 µL each of this dilution and was performed in triplicate. To discriminate between cell debris, dead cells, and living cells, gates of 5.88 µm/11.13 µm were used.

### 4.3. Proliferation Assay after Indirect Exposure

1 × 10^4^ endothelial cells were suspended in 800 µL culture media and transferred to the wells of a 24 well plate. Then, 200 µL culture medium was treated with CAP or carrier gas argon for 15 s (kINPen) or 30 s (MiniJet) in a separate 24-well plate and added to the cell suspension. Cells counts were performed at 4, 24, 48, 72, 96, and 120 h after the indirect CAP-exposure, as described in proliferation assay after direct CAP treatment.

### 4.4. MTT-Assay

2.0 × 10^4^ (4 h), 1.5 × 10^4^ (24 h), 1.0 ×x 10^4^ (48 h) and 6.5 × 10^3^ (72 h) cells were treated with CAP or carrier gas argon by kINPen (15 s) or Mini Jet (30 s) and were incubated for 4, 24, 48, and 72 h. To be able to normalize the metabolized MTT to the cell number, a second plate was planted out parallel. For performing the MTT-Assay, 20 µL MTT-Solution (MTT (3-(4,5-dimethylthiazol-2-yl)-2,5-diphenyltetrazolium bromide; Carl Roth, Karlsruhe, Germany) in a. bidest (5mg/mL) was added and incubated over 3 h. After incubation, the culture media were removed, and 120 µL lysis reagent (DMSO (Carl Roth, Karlsruhe, Germany) with 2.5% w/w SDS (Sodium Dodecyl Sulfate) (Carl Roth, Karlsruhe, Germany) and 150 µL 37% HCl (Carl Roth, Karlsruhe, Germany) were added and incubated over 10 min. Absorption at 565 nm was measured using an Infinite M200 plate reader (Tecan, Männedorf, Switzerland).

### 4.5. Glucose Metabolism-Assay

2.0 × 10^4^ cells were treated with 15 s CAP or carrier gas argon by kINPen and were incubated for 4, 24, 48, and 72 h. The glucose concentration of the cell-free supernatant was measured with a glucose detection kit (r-biopharm, Darmstadt, Germany) after the incubation. The number of viable cells was examined with CASY cell counter and analyzer. The glucose concentration was normalized to the cell count.

### 4.6. Caspase-3/7-Assay 

1.5 × 10^4^ (24 h), 1.0 × 10^4^ (48 h) and 6.5 × 10^3^ (72 h) cells were treated with CAP or argon for 15 s (kINPen) and 30 s (Mini Jet). As positive control cells were treated with cycloheximide (15 µM in cell culture medium; Carl Roth, Karlsruhe, Germany). To be able to normalize the measured fluorescence intensity to the cell number, a second plate was planted out parallel. After the incubation period, the used medium was removed, and 100 μL of Caspase 3/7 detection solution (CellEvent^TM^ Caspase 3/7 Green Detection Reagent (Thermo Fisher Scientific, Waltham, MA, USA) and 10 µM in DPBS with 5% FCS *v/v*) were incubated for 45 min. The fluorescence (535 nm) was measured after excitation (495 nm) using a plate reader. 

### 4.7. TUNEL-Assay

1.5 × 10^4^ (24 h), 1.0 × 10^4^ (48 h) and 6.5 × 10^3^ (72 h) cells were treated with CAP or argon for 15 s (kINPen) and 30 s (Mini Jet). Additionally, negative controls lacking fluorescent labeling and a nuclease-treated positive control were included. To be able to normalize the measured absorption to the cell number later, a second cell culture plate was simultaneously performed. The TiterTACS™ Colorimetric Apoptosis Detection Kit (Trevigen, Gaithersburg, MD, USA) was used according to the manufacturer’s instructions. Absorption was measured using the Infinite M200 plate reader (Tecan, Männedorf, Switzerland).

### 4.8. Migration-Assay

FluoroBlok Transwell inserts (Corning, New York, NY, USA; pore size 8 µm) were inserted into the wells of a 24-well cell culture plate. For the assay, 5.0 × 10^4^ cells were suspended in 200 μL culture media. The wells of the cell culture plate were filled with medium with or without VEGF (26.6 ng/mL). After 6 h of incubation (37 °C, 5% CO_2_), the insets were removed, washed twice with DPBS, and fixated with 100% Methanol. Cells were stained with DAPI (1 µg/mL) over 15 min at room temperature. The bottom membrane was cut out and transferred to microscope slides. The cover slips were mounted with 33% Glycerol. Edges were sealed with transparent nail varnish. With the BZ-II Analyzer software, the number of cells was determined. 

**Figure 8:**
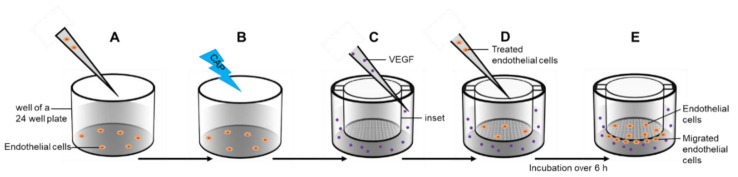
Migration assay. Endothelial cells were transferred in a well of a 24-well plate (**A**) and treated with cold atmospheric plasma (CAP) or argon carrier gas (**B**). Cell culture inserts were placed in another 24 well plates and a VEGF gradient was established over the membrane (**C**). Treated cells were transferred into the inset (**D**) and incubated over 6 h. Cells migrated through the membrane pores (**E**).

To investigate the influence of CAP on migration, cell suspensions were treated with CAP or argon carrier gas released by the kINPen over 15 s before seeding. The subsequent assay procedure remained unchanged. The assay setup is shown in Figure 8. 

### 4.9. Tube Formation-Assay

µ-Slides (ibidi, Gräfelfing, Germany) were coated with 10 µL Matrigel/Medium (5 mg/mL Matrigel (Corning, New York, NY, USA)/RPMI 1640 (PAN Biotech, Aidenbach, Germany) 1:1, *v/v*) per Well. In total, 4.0 × 10^4^ HDMEC were suspended in 200 µL medium and were treated with 15 s CAP or carrier gas argon only (as control treatment). Then, 50 µL cell suspension was transferred into the wells of the µ slides in triplicates. After an incubation period of 6 h at 37 C and 5% CO_2_ pictures were captured with Observer Z1 (Zeiss, Jena, Germany) with the software Zen 2012 pro (blue version, Zeiss, Jena, Germany). We analyzed the total tube length with imageJ (version 1.53e, NIH, Bethesda, MD, USA).

### 4.10. Hypoxanthine-Guanine-Phosphoribosyl-Transferase (HPRT) Assay

5 × 10^4^ CCL-93 cells were suspended in 200 µL media and treated with 10 s CAP or carrier gas argon. The cells were propagated in medium containing 6-thioguanin (10 µg/mL). As control CAP and carrier gas treated cells were also propagated in medium without 6-thioguanin 24, 48 and 72 h after CAP exposure, the number of viable cells were measured with CASY cell counter and analyzer, as described under point 4.2.

## 5. Conclusions

This study demonstrated the anti-proliferative effects of CAP on endothelial HDMEC, which depend on the duration of treatment and are due to the induction of apoptosis by CAP. In addition, it was shown that the migration of endothelial cells could be inhibited directly by CAP. The results of this work show that CAP is used to suppress angiogenesis and that tumor growth and the metastatic spread of malignant tumors can be directly influenced.

## Abbreviations

bFGF	basic fibroblast growth factor
CAP	cold atmospheric plasma
CTRL	control
ECGM-MV2	Endothelial Cell Growth Medium MV 2
Flt-1	fms related tyrosine kinase 1
HDMEC	Human Dermal Microvascular Endothelial Cells
KDR	kinase insert domain receptor
PDGF	platelet derivated growth factor
PECAM 1	platelet endothelial adhesion molecule 1
SDS	Sodium Dodecyl Sulfate
VE Cadherin	vascular endothelial cadherin
VEGF	vascular endothelial growth factor
